# Long noncoding RNA BSN-AS2 induced by E2F1 promotes spinal osteosarcoma progression by targeting miR-654-3p/SYTL2 axis

**DOI:** 10.1186/s12935-020-01205-y

**Published:** 2020-04-25

**Authors:** Xianwei Zhou, Jitian Li, Junyan Teng, Yufeng Liu, Di Zhang, Linyun Liu, Wenming Zhang

**Affiliations:** 1 Spine Surgery, Luoyang Orthopedic Hospital of Henan Province, No. 100 Yongping Road, Zhengzhou, 450000 Henan China; 2 Laboratory of Bone Tumor, Luoyang Orthopedic Hospital of Henan Province, Zhengzhou, 450000 Henan China; 3 Department of Osteoarthrosis & Health Management Center, Luoyang Orthopedic Hospital of Henan Province, Zhengzhou, 450000 Henan China

**Keywords:** BSN-AS2, E2F1, miR-654-3p, SYTL2, Spinal osteosarcoma

## Abstract

Spinal osteosarcoma (OS) is a rare and aggressive malignancy. Long noncoding RNA (lncRNA) BSN-AS2 has been shown to be an oncogenic gene in several cancers. However, the role and function of BSN-AS2 in spinal OS were unfamiliar. Our study identified that BSN-AS2 expression was boosted in spinal OS tissues and cell lines. Transcription factor E2F1 induced the upregulation of BSN-AS2 expression in spinal OS cells. Afterwards, loss-of-function assays indicated that BSN-AS2 depletion reduced cell proliferation, migration and invasion as well as promoted cell apoptosis in spinal OS. Thereafter, RIP, RNA pull down and luciferase reporter assays manifested BSN-AS2 could sponge miR-654-3p in spinal OS. After that, the binding effect of between miR-654-3p and SYTL2 was proved. Finally, rescue experiments illustrated that miR-654-3p inhibition or SYTL2 overexpression could counteract the inhibitory effect caused by BSN-AS2 deficiency on spinal OS progression. In conclusion, the availability of miR-654-3p was antagonized by E2F1-induced BSN-AS2 for SYTL2-meidated spinal OS progression.

## Background

Osteosarcoma (OS) is a common type of primary malignant tumor of bone. It is more frequently occurring in the extremities than in the spine [1–3]. OS mainly occurs in children and adolescents [4]. Primary OS of the spine is very rare, and among all spinal malignancies, spinal OS only accounts for 3–5% [5, 6]. As a result of its rarity, there is still limited case for successful treatment of spinal OS [7, 8]. Unfortunately, the high rates of recurrence, metastasis and mortality of spinal OS resulted in an extremely low 5-year survival rate of it [9, 10]. Therefore, exploring novel biomarkers participated in the pathogenesis of spinal OS is of great significance.

Long noncoding RNAs (lncRNAs) are a group of noncoding RNAs that generally are defined as RNA transcripts of over 200 nucleotides without protein coding ability [11]. Previously, researchers have identified that lncRNAs play a pivotal part in various biological processes, such as cell proliferation, invasion and migration [12–14]. Moreover, lncRNAs are largely found to be implicated with the development of cancers. For example, silence of lncRNA TUG1 inhibits the progression of thyroid cancer via targeting miR-145/ZEB1 axis [15]. LncRNA CCAL promotes the progression of colorectal cancer by suppression of activator protein 2α (AP-2α) to activate Wnt/β-catenin pathway [16]. LncRNA PEG10 is boosted in esophageal cancer tissues, and inhibition of PEG10 alleviates cell proliferation and invasion, and induces apoptosis in esophageal cancer [17]. Upregulation of lncRNA BCAR4 accelerates the malignant development of non-small cell lung cancer, indicating BCAR4 might serve as a potential biomarker for non-small cell lung cancer treatment [18]. In our research, lncRNA BSN-AS2 was found to aberrantly highly express in spinal OS tissues compared with adjacent normal tissues. Besides, BSN-AS2 differentially expressed in pheochromocytoma (PCC) samples and was recognized as an independent prognostic biomarker and potential therapeutic target for PCC [19]. Nevertheless, the underlying role and function of lncRNA BSN-AS2 in spinal OS was largely unknown. Additionally, BSN-AS2 is a newly-identified lncRNA and related research about its role in cancers is extremely limited. However, its abnormal upregulation in spinal OS tissues interested us.

The E2F transcription factors are vital participants in various cellular events in cancers [20]. Among the E2F family, E2F1 is the most thoroughly studied transcription factor in human malignancies, which exerts important effect in regulating cancer progression. For instance, transcription factor E2F1 aggravates EMT progression in small cell lung cancer via modulating ZEB2 [21]. Transcription factor E2F1 regulates proliferative and apoptotic functions in hepatocellular carcinoma [22]. However, the function of E2F1 in spinal OS still needs to be clarified.

In our study, we focused on investigating the role of BSN-AS2 in spinal OS, and our results might provide some inspirations for finding an underlying biomarker for spinal OS treatment.

## Methods

### Tissue samples

50 matched samples of osteosarcoma tissues and adjacent normal tissues were collected under the approval from the Ethical Committee of Luoyang Orthopedic Hospital of Henan Province. In the present study, all patients signed the informed consent forms and none had treated by chemotherapy or radiotherapy before study. All tissue specimens were frozen in liquid nitrogen instantly after surgical resection, and then stored at −80 °C until RNA extraction.

### Cell culture

Human osteoblasts (hFOB) and OS cells (U2OS, Saos-2, MG-63 and SW1353) were bought from Chinese Academy of Sciences (Beijing, China), and cultured in DMEM (Invitrogen, MA, USA) containing 10% fetal bovine serum (FBS; Invitrogen), 1% penicillin/streptomycin (Sigma-Aldrich, Milan, Italy). Besides, an incubator of 5% CO_2_ at 37 °C was applied.

### Cell transfection

U2OS and Saos-2 cells were transfected with specific shRNAs against E2F1 (sh-E2F1), BSN-AS2 (sh-BSN-AS2#1#2#3), SYTL2 (sh-SYTL2) and the negative control (sh-NC), and pcDNA3.1/E2F1, pcDNA3.1/SYTL2 and the empty pcDNA3.1 vector (GenePharma, Shanghai, China). The miR-654-3p mimics and NC mimics were gained from GenePharma. Each plasmid was transfected into cells via Lipofectamine 2000 (Invitrogen). Related sequences were provided in Table 1.

**Table 1:** The sequences of gene overexpression and interference were presented

shRNA	Sequences (5′-3′)
sh-NC	CCGGACGTGTGCCGAGTCTCTATGGTCTCGAGTGCACACGGCTCAGAGATACCATTTTTG
sh-BSN-AS2#1	CCGGCTTCGGTCGACCCTCCGGTGGACTCGAGGAAGCCAGCTGGGAGGCCACCTTTTTTG
sh-BSN-AS2#2	CCGGATGAAGGGGCGAGGGAGTGTAACTCGAGTACTTCCCCGCTCCCTCACATTTTTTTG
sh-BSN-AS2#3	CCGGACGACTATCGGTCGGACGACTCTCGAGTGCTGATAGCCAGCCTGCTGATTTTTTTG
sh-NC	CCGGATATTGATCAGTCATTAGATGTCTCGAGTATAACTAGTCAGTAATCTACATTTTTG
sh-E2F1	CCGGGAGACACGAGACGCGGAGTCGACTCGAGCTCTGTGCTCTGCGCCTCAGCTTTTTTG
sh-NC	CCGGACAGAAGATGTCGGTTGGATGTCTCGAGTGTCTTCTACAGCCAACCTACATTTTTG
sh-SYTL2	CCGGTCGATATTTGATGTGCTCACCCCTCGAGAGCTATAAACTACACGAGTGGGTTTTTG

mimics	Sequences (5′-3′)
NC mimics	GCUGCUGAAUCAUUAUCCCCUU
miR-654-3p mimics	UGGUUUACCGUCCCACAUACAU

Inhibitor	Sequences (5′-3′)
NC inhibitor	AAGUCAGGUGAUGGACAGCAUA
miR-654-3p inhibitor	AAGGUGAUGGUCAGCAGACAUA

pcDNA3.1	Sequences (5′-3′)
pcDNA3.1/E2F1	ATGGCCTTGGCCGGGGCCCCTGCGGGCGGCCCATGCGCGCCGGCGCTGGAGGCCCTGCTCGGGGCCGGCGCGCTGCGGCTGCTCGACTCCTCGCAGATCGTCATCATCTCCGCCGCGCAGGACGCCAGCGCCCCGCCGGCTCCCACCGGCCCCGCGGCGCCCGCCGCCGGCCCCTGCGACCCTGACCTGCTGCTCTTCGCCACACCGCAGGCGCCCCGGCCCACACCCAGTGCGCCGCGGCCCGCGCTCGGCCGCCCGCCGGTGAAGCGGAGGCTGGACCTGGAAACTGACCATCAGTACCTGGCCGAGAGCAGTGGGCCAGCTCGGGGCAGAGGCCGCCATCCAGGAAAAGGTGTGAAATCCCCGGGGGAGAAGTCACGCTATGAGACCTCACTGAATCTGACCACCAAGCGCTTCCTGGAGCTGCTGAGCCACTCGGCTGACGGTGTCGTCGACCTGAACTGGGCTGCCGAGGTGCTGAAGGTGCAGAAGCGGCGCATCTATGACATCACCAACGTCCTTGAGGGCATCCAGCTCATTGCCAAGAAGTCCAAGAACCACATCCAGTGGCTGGGCAGCCACACCACAGTGGGCGTCGGCGGACGGCTTGAGGGGTTGACCCAGGACCTCCGACAGCTGCAGGAGAGCGAGCAGCAGCTCCAGCGCCTGGCCTACGTGACGTGTCAGGACCTTCGTAGCATTGCAGACCCTGCAGAGCAGATGGTTATGGTGATCAAAGCCCCTCCTGAGACCCAGCTCCAAGCCGTGGACTCTTCGGAGAACTTTCAGATCTCCCTTAAGAGCAAACAAGGCCCGATCGATGTTTTCCTGTGCCCTGAGGAGACCGTAGGTGGGATCAGCCCTGGGAAGACCCCATCCCAGGAGGTCACTTCTGAGGAGGAGAACAGGGCCACTGACTCTGCCACCATAGTGTCACCACCACCATCATCTCCCCCCTCATCCCTCACCACAGATCCCAGCCAGTCTCTACTCAGCCTGGAGCAAGAACCGCTGTTGTCCCGGATGGGCAGCCTGCGGGCTCCCGTGGACGAGGACCGCCTGTCCCCGCTGGTGGCGGCCGACTCGCTCCTGGAGCATGTGCGGGAGGACTTCTCCGGCCTCCTCCCTGAGGAGTTCATCAGCCTTTCCCCACCCCACGAGGCCCTCGACTACCACTTCGGCCTCGAGGAGGGCGAGGGCATCAGAGACCTCTTCGACTGTGACTTTGGGGACCTCACCCCCCTGGATT TCTGA
pcDNA3.1/SYTL2	ATGAGAAAGTCTGTTCCAGCATTTCTCCAAGATGAGAGTGATGACAGAGAAACAGATACAGCATCAGAAAGCAGTTACCAGCTCAGCAGACACAAGAAGAGCCCGAGCTCTTTAACCAATCTTAGCAGCTCCTCTGGCATGACGTCCTTGTCTTCTGTGAGTGGCAGTGTGATGAGTGTTTATAGTGGAGACTTTGGCAATCTGGAAGTTAAAGGAAATATTCAGTTTGCAATTGAATATGTGGAGTCACTGAAGGAGTTGCATGTTTTTGTGGCCCAGTGTAAGGACTTAGCAGCAGCGGATGTAAAAAAACAGCGTTCAGACCCATATGTAAAGGCCTATTTGCTACCAGACAAAGGCAAAATGGGCAAGAAGAAAACACTCGTAGTGAAGAAAACCTTGAATCCTGTGTATAACGAAATACTGCGGTATAAAATTGAAAAACAAATCTTAAAGACACAGAAATTGAACCTGTCCATTTGGCATCGGGATACATTTAAGCGCAATAGTTTCCTAGGGGAGGTGGAACTTGATTTGGAAACATGGGACTGGGATAACAAACAGAATAAACAATTGAGATGGTACCCTCTGAAGCGGAAGACAGCACCAGTTGCCCTTGAAGCAGAAAACAGAGGTGAAATGAAACTAGCTCTCCAGTATGTCCCAGAGCCAGTCCCTGGTAAAAAGCTTCCTACAACTGGAGAAGTGCACATCTGGGTGAAGGAATGCCTTGATCTACCACTGCTAAGGGGAAGTCATCTAAATTCTTTTGTTAAATGTACCATCCTTCCAGATACAAGTAGGAAAAGTCGCCAGAAGACAAGAGCTGTAGGGAAAACCACCAACCCTATCTTCAACCACACTATGGTGTATGATGGGTTCAGGCCTGAAGATCTGATGGAAGCCTGTGTAGAGCTTACTGTCTGGGACCATTACAAATTAACCAACCAATTTTTGGGAGGTCTTCGTATTGGCTTTGGAACAGGTAAAAGTTATGGGACTGAAGTGGACTGGATGGACTCTACTTCAGAGGAAGTTGCTCTCT GGGAGAAGATGGTAAACTCCCCCAATACTTGGATTGAAGCAACACTGCCTCTCAGAATGCTTTTGATTGC CAAGATTTCC AAATTGA

### Quantitative real-time PCR (qRT-PCR)

Total RNA was extracted from cultured cells utilizing TRIzol reagent (Invitrogen) and then was reverse transcribed into cDNA using Reverse Transcription Kit (Takara, Dalian, China). qRT-PCR was progressed by SYBR-Green Real-Time PCR Kit (Takara). Relative expression quantity was calculated with usage of 2^−ΔΔCT^ method, and normalized to GAPDH/U6. Related primer sequences were provided in Table 2.

**Table 2:** The sequences of gene primer were presented

Genes	Primer sequences (5′-3′)
BSN-AS2	F: GCAGGCGTCATAAGGACAGG
	R: TGCGTCTCTGAATACACTTGTTC
miR-654-3p	F: CCGAGTATGTCTGCTGACCAT
	R: CTCAACTGGTGTCGTGGA
miR-515-5p	F: GCCGATTCTCCAAAAGAAAGCAC
	R: CTCAACTGGTGTCGTGGA
miR-219a-5p	F: CCGAGTGATTGTCCAAACG
	R: CTCAACTGGTGTCGTGGA
miR-4782-3p	F: GCCGAGTGATTGTCTTCATATC
	R: CTCAACTGGTGTCGTGGA
miR-6766-3p	F: CCGAGGATTGTCTTCCCCCA
	R: CTCAACTGGTGTCGTGGA
miR-2355-3p	F: GCCGAGATTGTCCTTGCTGTT
	R: CTCAACTGGTGTCGTGGA
E2F1	F: ACGCTATGAGACCTCACTGAA
	R: TCCTGGGTCAACCCCTCAAG
SYTL2	F: GCCCAGTGTAAGGACTTAGCA
	R: GCCTTTGTCTGGTAGCAAATAGG
CLN8	F: TGGTCGCTGGCTTTGTCTTC
	R: AGAACGGTAAGTGGCATTCAG
GAPDH	F: GGAGCGAGATCCCTCCAAAAT
	R: GGCTGTTGTCATACTTCTCATGG
U6	F: CTCGCTTCGGCAGCACA
	R: AACGCTTCACGAATTTGCGT

### ChIP assay

U2OS and Saos-2 cells were cross-linked with formaldehyde (Sigma-Aldrich) for 10 min and the reaction was terminated by using glycine treatment for 10 min. The extracted chromatin was sonicated and fragmented into 150–900 bp. Anti-E2F1 (ab179445; Abcam, Cambridge, MA, USA; 1/1000) and anti-IgG (#2729; Cell Signaling Technology, Danvers, MA, USA; 1/1000) were used to be immunoprecipitated with chromatin fragments. After cleaning, elution, and de-crosslinking, qRT-PCR was performed.

### Western blot

Protein was obtained from cells with RIPA lysis buffer (Beyotime, Shanghai, China) supplied with protease inhibitors. Protein was separated through SDS-PAGE and moved to PVDF membranes (Millipore, Darmstadt, Germany). After being sealed with 5% skimmed milk, the membranes were cultivated with primary antibodies for E2F1 (ab218527, 1/1000), SYTL2 (ab231133, 1/1000), Akt (ab235958, 1/1000), p-Akt (ab81283, 1/5000), ERK (ab54230, 1/1000), p-ERK (ab201015, 1/1000) and GAPDH (ab8245, 1/1000) from Abcam (Cambridge, USA). Secondary antibodies were added for cultivating for 1 h. The amount of protein was examined via chemiluminescence detection system.

### CCK-8 assay

1 × 10^3^ cells were plated into fresh 96-well plates and cultured over specific time points. Ten microliters of CCK8 reagent were added to incubate for additional 4 h. The absorbance at 450 nm was determined under a microplate reader (Olympus, Tokyo, Japan).

### Colony formation assay

Transfected cells (1 × 10^3^) were cultured in 6-well plates and incubated for 2 weeks. After rinsing by PBS (Solarbio, Beijing, China), formaldehyde or crystal violet (Sigma-Aldrich) was applied for fixation or coloration, severally. Visible colonies were counted via a microscope (Olympus).

### Apoptosis assay

Briefly, cells were incubated in 6-well plates for 48 h and then being cleaned by PBS. After that, cells were fixed with 70% ice‐cold ethanol (Sigma-Alidrich) for 2 h and then double-stained with propidium iodide and Annexin V‐fluorescein isothiocyanate. Apoptosis rate was examined by flow cytometer (BD Biosciences, Beijing, China).

### Luciferase reporter assay

The pGL3-BSN-AS2 promoter (Promega, MA, USA) was transfected with pcDNA3.1/E2F1 and pcDNA3.1 vector or sh-E2F1 and sh-NC into U2OS or Saos-2 cells. The wild-type (WT) and mutant (Mut) binding sites of BSN-AS2 sequence or SYTL2 3′UTR was sub-cloned into pmirGLO luciferase vector (Promega) to construct BSN-AS2-Wt/Mut or SYTL2-Wt/Mut, then co-transfected severally with miR-654-3p mimics or NC mimics into U2OS and Saos-2 cells. The luciferase activity was detected using Dual-Luciferase Reporter Assay System (Promega).

### TUNEL assay

TUNEL assay was conducted for measuring the fragmented DNA of apoptotic cells. 1 × 10^5^ U2OS and Saos-2 cells were plated in 24-well plates, and were fixed in paraformaldehyde. The nuclei were stained by the use of DAPI for 10 min. Finally, the numbers of TUNEL positive cells were photographed via a fluorescence microscope (Olympus).

### Transwell assay

The capacities of cell migration and invasion were measured on Transwell chambers (8 μm pore size, BD Biosciences). Transwell chamber was pre-coated with Matrigel (BD Biosciences) was employed for invasion assay and without Matrigel for migration assay. Cells (5 × 10^4^) in serum-free medium were placed onto the top compartment, while the bottom compartment containing 10% FBS was regarded as growth medium. After incubation, cells were fixed by using paraformaldehyde and then dyed with crystal violet. The number of migratory and invasive cells was counted by a microscope (Olympus).

### Subcellular fractionation

Nuclear/cytoplasmic fractionation PARIS Kit (Life Technologies, Carlsbad, CA) was used for collecting fractions of nuclear and cytoplasmic. qRT-PCR was performed to determine the relative expression of BSN-AS2, and U2 or GAPDH was nuclear control or cytoplasmic control.

### RNA pull down assay

The miR-654-3p-WT, miR-654-3p-Mut and NC were biotin labeled into Bio-miR-654-3p-WT, Bio-miR-654-3p-Mut and Bio-NC, respectively. Streptavidin-coated magnetic beads were applied for incubation of U2OS and Saos-2 cells. Pull-down assay was carried out in biotin-coupled RNA complex. The abundance of BSN-AS2 or SYTL2 in bound fractions was calculated in accordance with the results of qRT-PCR. In addition, cell protein lysates were mixed with the magnetic beads and biotin-labeled probes for BSN-AS2, finally analyzed by qRT-PCR.

### RIP assay

Supplier’s protocol of the EZMagna RIP Kit (Millipore) was strictly obeyed. Cells were lysed with RIP lysis buffer. Cell extracts were co-cultured with anti-Ago2 or anti-IgG conjugated with magnetic beads for 48 h. After purification, the relative expression levels of BSN-AS2, miR-654-3p and SYTL2 were evaluated by qRT-PCR analysis.

### Animal studies

Subcutaneous xenograft assay was undertaken using 6-week-old male BALB/C nude mice (Beijing Vital River Laboratory Animal Technology, Beijing, China), under the approval from the Animal Research Ethics Committee of Luoyang Orthopedic Hospital of Henan Province. 1 × 10^6^ U2OS cells transfected with sh-NC, sh-BSN-AS2#1 or sh-BSN-AS2#1 + SYTL2 were collected and injected subcutaneously into nude mice for 28 days. The volume of tumors was monitored every 4 days and calculated as 1/2 length × width^2^. After killing mice, the xenograft tumors were excised carefully for weigh assessment.

### Statistical analysis

Statistical analysis was completed by GraphPad Prism 7.0 software (La Jolla, CA, USA). Numerical data were manifested as mean ± SD. Differences among groups were compared via Student’s *t* test and one-way ANOVA. P < 0.05 had statistical significance. All experiments were done thrice independently at least.

## Results

**Fig. 1:**
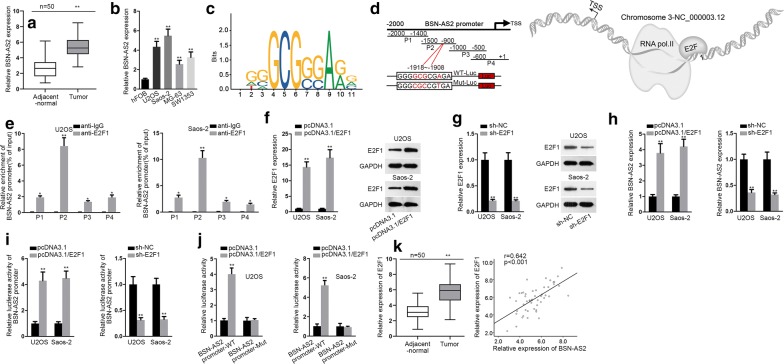
BSN-AS2 was transcriptionally activated by E2F1 in spinal OS. **a** The expression of BSN-AS2 in spinal OS tissues and adjacent-normal tissues was measured by qRT-PCR. **b** qRT-PCR revealed BSN-AS2 expression in spinal OS cells and hFOB cells. **c** JASPAR database presented the binding motif of E2F1 and the binding area of E2F1 on BSN-AS2 promoter. **d** The schematic diagram about role of E2F1 in BSN-AS2 transcription. **e** ChIP assay illustrated the binding facts between E2F1and BSN-AS2 promoter in part 2 (P2). **f**–**g** qRT-PCR and western blot assays detected the mRNA and protein expressions of E2F1. **h** qRT-PCR assessed the expression of BSN-AS2 in response to E2F1 upregulation or downregulation. **i** Luciferase reporter assays demonstrated the effects of E2F1 expression modulation on BSN-AS2 transcription. **j** Luciferase reporter assays verified binding facts between E2F1 and BSN-AS2 promoter in P2. **k** The expression of E2F1 in spinal OS tissues and adjacent-normal tissues was measured by qRT-PCR (left); Pearson correlation analysis illustrated the correlation between E2F1 and BSN-AS2 (right). ^*^P < 0.05, ^**^P < 0.01

Based on the results of qRT-PCR, BSN-AS2 was significantly upregulated in spinal OS tissues in comparison with adjacent-normal tissues (Fig. 1a). Also, BSN-AS2 expression was evidently upregulated in spinal OS cells compared with hFOB cells (Fig. 1b). These findings implied that BSN-AS2 was implicated with the development of spinal OS. Furthermore, the mechanism associated with the upregulation of BSN-AS2 was investigated in U2OS and Saos-2 cells, which presented higher expression of BSN-AS2. According to UCSC (http://genome.ucsc.edu/), the potential transcription factors of BSN-AS2 were identified. Among them, E2F1 was previously confirmed to be a transcription factor in cancers [23, 24]. Then by use of JASPAR database (http://jaspar.genereg.net/), the binding motif of E2F1 and BSN-AS2 promoter were found (Fig. 1c, left). And part 2 (P2) was predicted as the specific binding area of E2F1 on BSN-AS2 promoter (Fig. 1c, right). The schematic diagram about that E2F1 promoted the transcription of BSN-AS2 was shown in Fig. 1d. ChIP assay illustrated the strong affinity of E2F1 to P2 of BSN-AS2 promoter (Fig. 1e). Then E2F1 was effectively overexpressed and knocked down in U2OS and Saos-2 cells (Fig. 1f, g). Moreover, BSN-AS2 was positively regulated by E2F1. E2F1 upregulation or downregulation increased or decreased the expression of BSN-AS2 (Fig. 1h). Luciferase reporter assays demonstrated that overexpression of E2F1 increased the luciferase activity of BSN-AS2 promoter while ablation of E2F1 decreased that of BSN-AS2 promoter (Fig. 1i), indicating E2F1 transcriptionally upregulated BSN-AS2 in spinal OS. In addition, luciferase reporter assays further confirmed that the binding facts between BSN-AS2 promoter and E2F1 in P2 (− 1918 ~ − 1908) (Fig. 1J). Furthermore, E2F1 possessed higher level in spinal OS tissues than adjacent-normal tissues, and E2F1 was positively correlated with BSN-AS2 with regards the expression in tissues (Fig. 1k). To sum up, BSN-AS2 induced by E2F1 was upregulated in spinal OS tissues and cells.

**Fig. 2:**
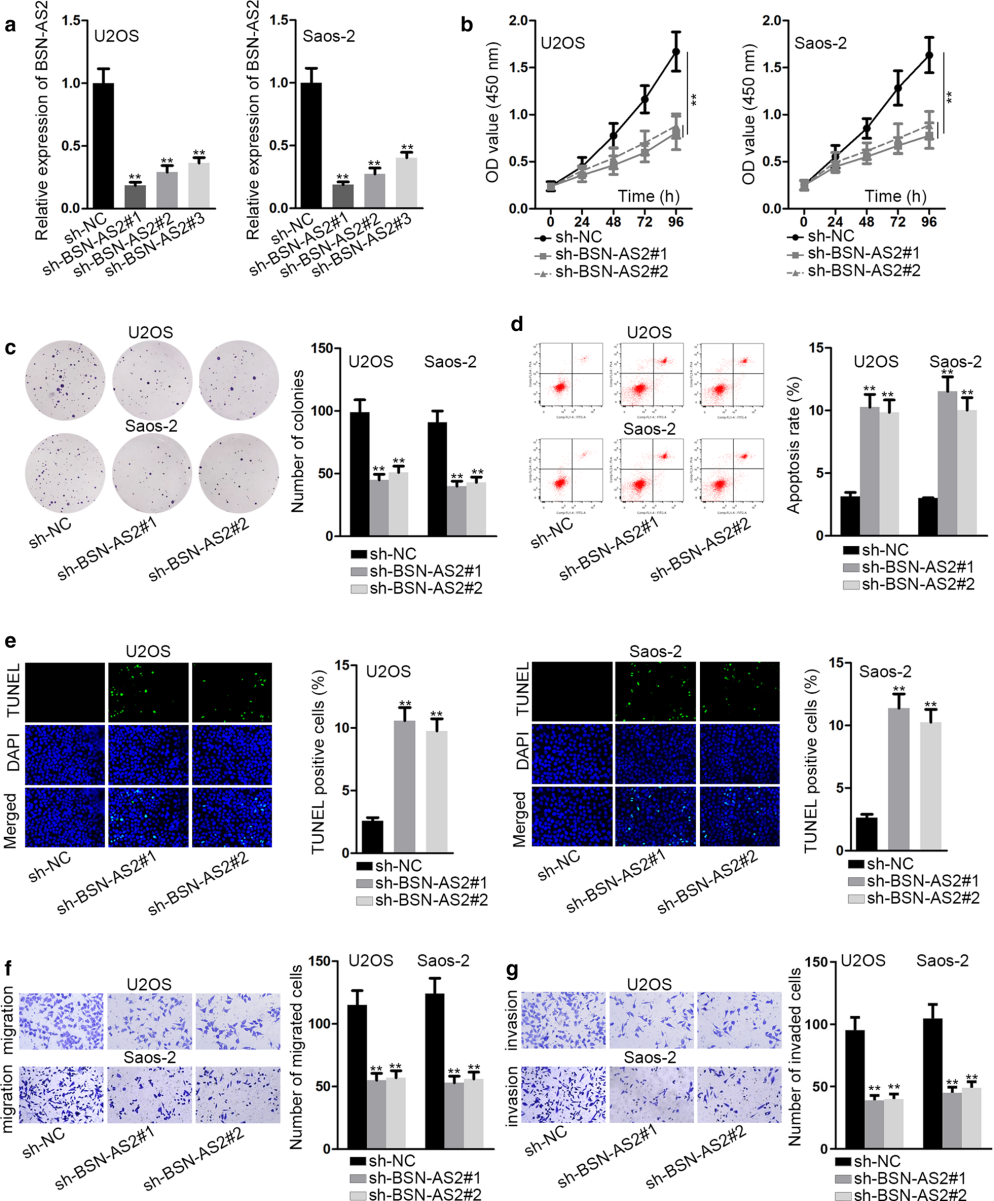
Depletion of BSN-AS2 inhibited spinal OS progression. **a** qRT-PCR disclosed BSN-AS2 was silenced in U2OS and Saos-2 cells. **b**, **c** CCK-8 and colony formation assays measured proliferation of U2OS and Saos-2 cells when knocking down BSN-AS2. **d**, **e** Flow cytometry analysis and TUNEL assay detected the apoptosis in BSN-AS2 silenced U2OS and Saos-2 cells. **f**, **g** Transwell assays evaluated cell migration and invasion in response to BSN-AS2 deficiency. ^**^P < 0.01

In order to explore the role of BSN-AS2 in spinal OS, a string of loss-of-function assays were undertaken in U2OS and Saos-2 cells. To begin with, BSN-AS2 was silenced in U2OS and Saos-2 cells by transfection with sh-BSN-AS2#1, sh-BSN-AS2#2 and sh-BSN-AS2#3 vectors (Fig. 2a). qRT-PCR disclosed that sh-BSN-AS2#1 and sh-BSN-AS2#2 could be selected for further study as they presented more satisfactory knockdown efficiency. CCK-8 and colony formation assays showed that BSN-AS2 descent markedly reduced the proliferation of U2OS and Saos-2 cells (Fig. 2b, c). Flow cytometry analysis and TUNEL assay demonstrated that knockdown of BSN-AS2 had positive effects on cell apoptosis in U2OS and Saos-2 cells (Fig. 2d, e). Next, transwell assays were implemented and disclosed cell migration and invasion were consistently suppressed due to BSN-AS2 deficiency (Fig. 2f, g). Besides, the role of AKT/ERK in cancers including OS has been extensively illustrated [25, 26]. In this research, when downregulating BSN-AS2, western blot assay measured that the expression of AKT- and ERK- related proteins presented no changes (Additional file 1: Fig. S1A). In summary, depletion of BSN-AS2 inhibited spinal OS progression.

**Fig. 3:**
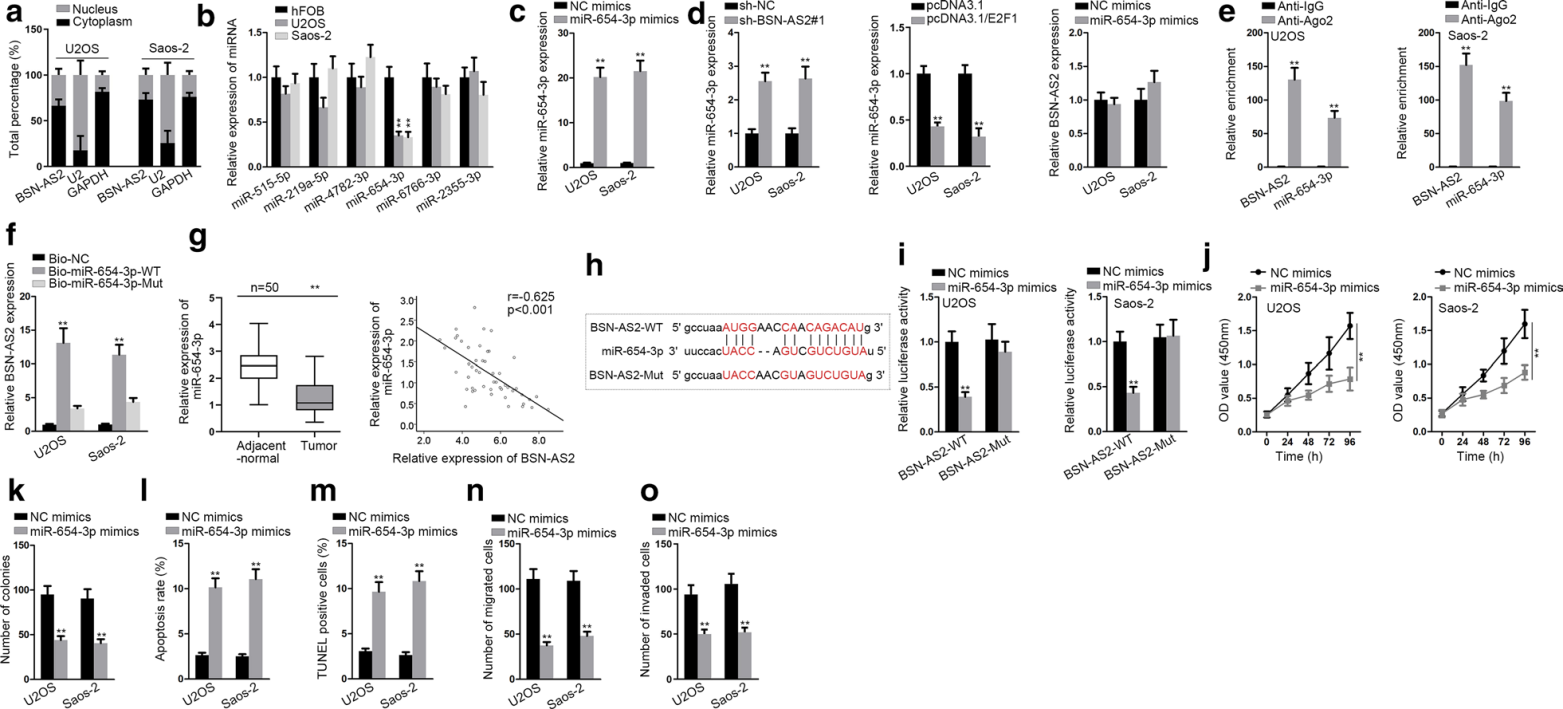
BSN-AS2 could bind with miR-654-3p in spinal OS. **a** Nuclear-cytoplasmic fractionation assay disclosed BSN-AS2 expression was enriched in cytoplasm. **b** qRT-PCR unveiled the expression of 6 miRNAs in hFOB, U2OS and Saos-2 cells. **c** qRT-PCR manifested the expression of miR-654-3p in miR-654-3p mimics transfected cells. **d** qRT-PCR testified the expression of miR-654-3p when downregulating BSN-AS2 or upregulating E2F1 as well as the expression of BSN-AS2 when overexpressing miR-654-3p. **e** RIP assay showed the enrichment of BNS-AS2 and miR-654-3p in anti-Ago2 or anti-IgG group. **f** RNA pull down assay revealed BSN-AS2 could bind with miR-654-3p. **g** The expression of miR-654-3p in spinal OS tissues and adjacent-normal tissues was measured by qRT-PCR (left); Pearson correlation analysis illustrated the correlation between miR-654-3p and BSN-AS2 (right). **h** The putative binding sites between BSN-AS2 and miR-654-3p. **i** Luciferase reporter assay uncovered BSN-AS2 could bind with miR-654-3p. **j**, **k** CCK-8 and colony formation assays measured cell proliferation in response to miR-654-3p overexpression. **l**, **m** Flow cytometry analysis and TUNEL assay detected cell apoptosis in miR-654-3p mimics transfected cells. **n**, **o** Transwell assays illustrated cell migration and invasion when overexpressing miR-654-3p. ^**^P < 0.01

Thereafter, the downstream regulatory mechanism of BSN-AS2 was probed. Nuclear-cytoplasmic fractionation assay disclosed BSN-AS2 expression was enriched in cytoplasm (Fig. 3a), suggesting that BSN-AS2 might function as a ceRNA to regulate gene expression at post-transcriptional level. Based on starBase database (http://starbase.sysu.edu.cn/), 10 miRNAs were screened out for that they possessed binding sites for BSN-AS2, and RNA pull down assay examined that 6 miRNAs were enriched in biotin BSN-AS2 probe group (Additional file 1: Fig. S1B). Afterwards, qRT-PCR measured the expression of 6 pre-qualified miRNAs in spinal OS cells (U2OS and Saos-2) and control cells. Among them miR-654-3p was dramatically downregulated in U2OS and Saos-2 cells (Fig. 3b). Furthermore, we overexpressed miR-654-3p by transfecting miR-654-3p mimics vector, disclosing a strikingly enhancement of miR-654-3p in U2OS and Saos-2 cells (Fig. 3c). qRT-PCR demonstrated that BSN-AS2 silence increased the expression of miR-654-3p and E2F1 overexpression decreased that of miR-654-3p, but miR-654-3p overexpression had no significant effect on BSN-AS2 expression (Fig. 3d), indicating that BSN-AS2 might regulate miR-654-3p in spinal OS. RIP assay showed BNS-AS2 and miR-654-3p were accumulated in anti-Ago2 group rather than anti-IgG group (Fig. 3e). RNA pull down assay revealed BSN-AS2 could be pulled down by bio-miR-654-WT rather than bio-miR-654-Mut and bio-NC (Fig. 3f). Similarly, qRT-PCR detected that miR-654-3p was obviously downregulated in spinal OS tissues, and miR-654-3p was negatively correlated with BNS-AS2 (Fig. 3g). Next, we obtained the putative binding sites between BSN-AS2 and miR-654-3p (Fig. 3h). Luciferase reporter assay uncovered that miR-654-3p upregulation efficiently decreased the luciferase activity of BSN-AS2-WT but not that of BSN-AS2-Mut (Fig. 3i). Thus, the interaction of BSN-AS2 and miR-654-3p in spinal OS was confirmed. After that, the function of miR-654-3p in spinal OS was determined. According to CCK-8 and colony formation assays, cell proliferation was impeded owing to miR-654-3p overexpression (Fig. 3j, k). Reversely, cell apoptosis was promoted by miR-654-3p upregulation (Fig. 3l, m). Cell migration and invasion were alleviated as a result of the transfection of miR-654-3p mimics (Fig. 3n, o). Moreover, AKT and ERK pathways were not affected by miR-654-3p upregulation (Additional file 1: Fig. S1C). All in all, BSN-AS2 sponged with miR-654-3p, which exerted suppressive functions on spinal OS progression.

**Fig. 4:**
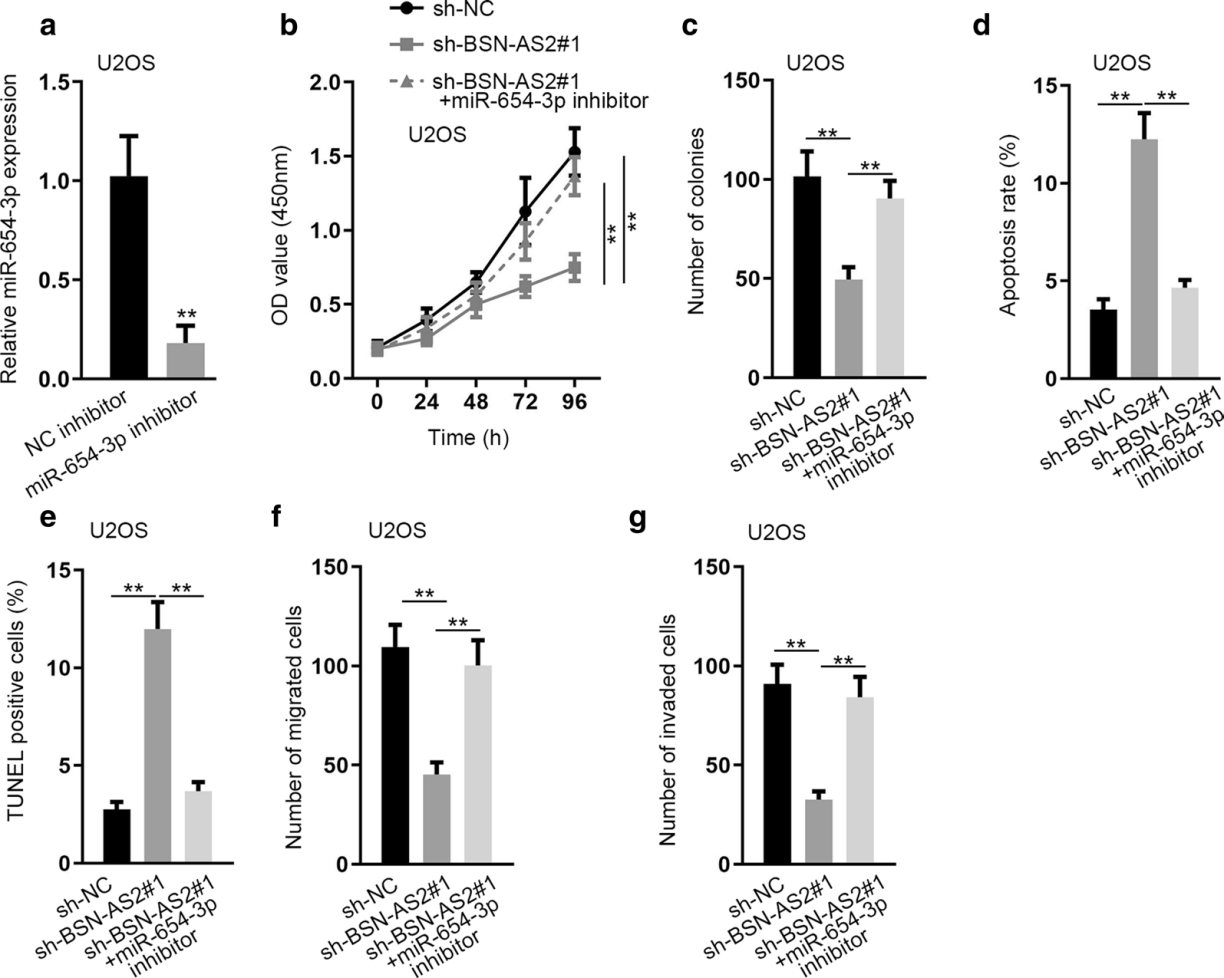
BSN-AS2 promoted spinal OS progression via inhibiting miR-654-3p expression. **a** qRT-PCR measured miR-654-3p expression in miR-654-3p inhibitor transfected cells. **b**, **c** CCK-8 and colony formation assays measured cell proliferation in transfected groups. **d**, **e** Flow cytometry analysis and TUNEL assay detected cell apoptosis in transfected groups. **f**, **g** Transwell assays assessed cell migration and invasion in transfected groups. ^**^P < 0.01

Therefore, we performed several rescue experiments to illustrate the role of BSN-AS2/miR-654-3p axis in spinal OS. Firstly, the inhibitory efficiency of miR-654-3p was assessed in miR-654-3p inhibitor transfected cells (Fig. 4a). Then CCK-8 and colony formation assays proved that miR-654-3p inhibition rescued the inhibited cell proliferation caused by BSN-AS2 insufficiency (Fig. 4b, c). Flow cytometry analysis and TUNEL assay found that miR-654-3p downregulation reversed the promoting function of BSN-AS2 depletion on cell apoptosis (Fig. 4d, e). Transwell assays also confirmed the rescuing role of miR-654-3p inhibitor in BSN-AS2 silence-mediated function on cell migration and invasion (Fig. 4f, g). Finally, western blot assay demonstrated that the expression of AKT/ERK pathway-associated proteins was not changed under the indicated transfection conditions (Additional file 1: Fig. S1D). In a summary, BSN-AS2 promoted spinal OS progression via inhibiting miR-654-3p expression.

**Fig. 5:**
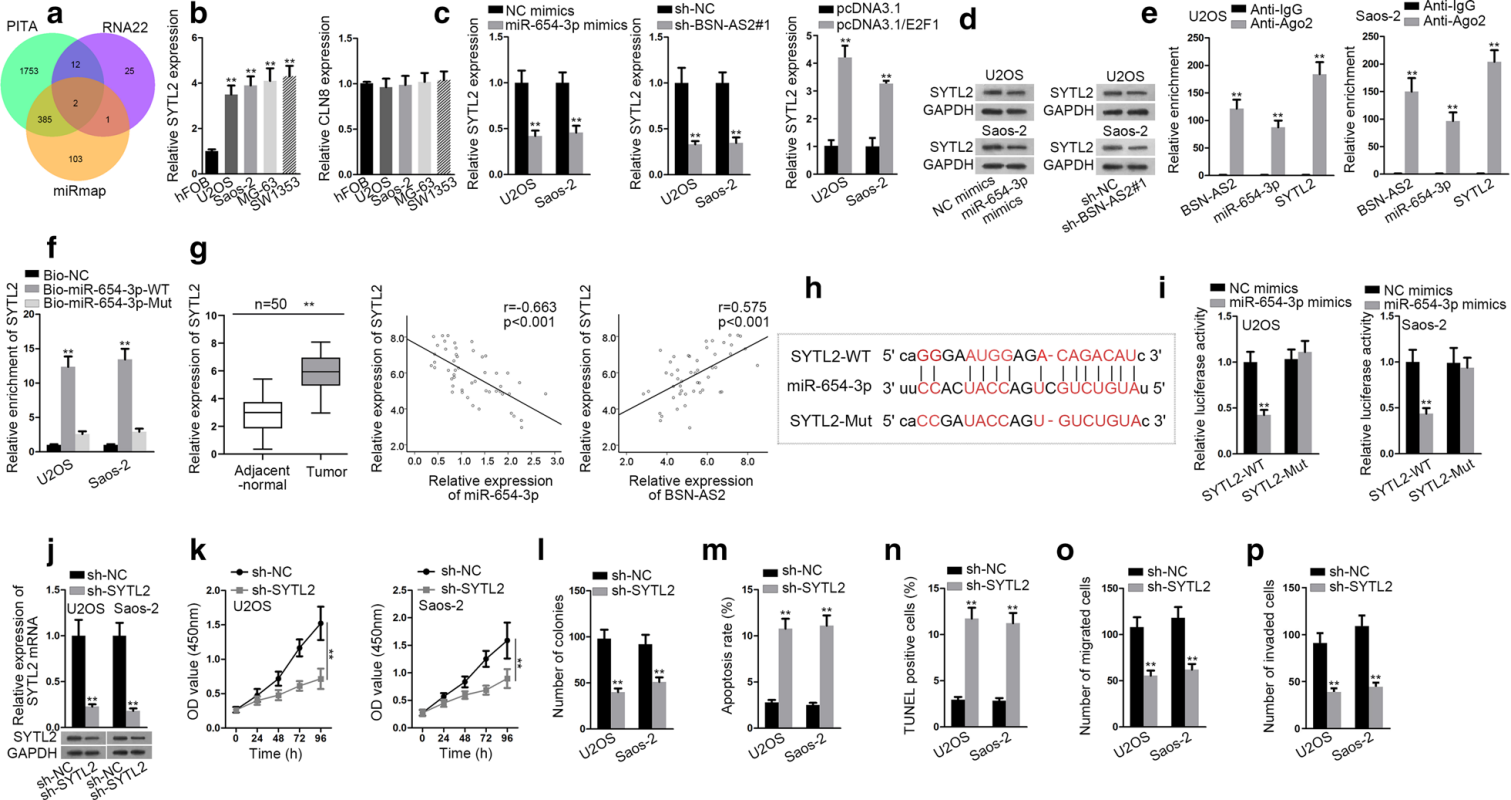
MiR-654-3p targeted SYTL2 to regulate spinal OS progression. **a** StarBase showed SYTL2 and CLN8 were potentially targeted by miR-654-3p. **b** qRT-PCR indicated the expression of SYTL2 and CLN8 in spinal OS cells and hFOB cells. **c**, **d** qRT-PCR and western blot assays uncovered the mRNA and protein expressions of STYL2. **e** RIP assay manifested the enrichment of BSN-AS2, miR-654-3p and SYTL2 in anti-Ago2 group. **f** RNA pull down showed miR-654-3p could bind with SYTL2. **g** The expression of SYTL2 in spinal OS tissues and adjacent-normal tissues was measured by qRT-PCR (left); Pearson correlation analysis illustrated the correlation between SYTL2 and miR-654-3p/BSN-AS2 (middle and right). **h** The potential binding site for miR-654-3p and SYTL2 was exhibited. **i** Luciferase reporter assay illustrated miR-654-3p could target SYTL2 in spinal OS. **j** qRT-PCR and western blot assays detected the mRNA and protein expressions of SYTL2. **k**, **l** CCK-8 and colony formation assays showed cell proliferation when inhibiting SYTL2. **m**, **n** Flow cytometry analysis and TUNEL assay determined cell apoptosis in SYTL2 silenced cells. **o**, **p** Transwell assay demonstrated cell migration and invasion in response to SYTL2 attenuation. ^**^P < 0.01

Further, the downstream mRNAs of miR-654-3p in spinal OS were investigated. SYTL2 and CLN8 were potentially targeted by miR-654-3p based on starBase (Fig. 5a). Then the expression of SYTL2 and CLN8 in spinal OS cells and hFOB cells was detected by qRT-PCR. The results showed SYTL2 expression was predominantly increased in spinal OS cells (Fig. 5b). Hence, we speculated miR-654-3p might target SYTL2 to regulate spinal OS progression. Moreover, the mRNA and protein expressions of STYL2 were attenuated in response to miR-654-3p overexpression or BSN-AS2 suppression, but upregulated in response to E2F1 overexpression (Fig. 5c, d). RIP assay manifested BSN-AS2, miR-654-3p and SYTL2 were enriched in Ago2-containing beads (Fig. 5e). Besides, SYTL2 was enriched in bio-miR-654-3p-WT group instead of bio-miR-654-3p-Mut and bio-NC groups (Fig. 5f). In a similar way, qRT-PCR measured that SYTL2 was remarkably upregulated in spinal OS tissues compared with control group. Besides, SYTL2 was negatively correlated with miR-654-3p, but positively correlated with BSN-AS2 (Fig. 5g). Then the potential binding site for miR-654-3p and SYTL2 was exhibited in Fig. 5h. Furthermore, the luciferase activity of the SYTL2-WT but not that of SYTL2-Mut was decreased by upregulation of miR-654-3p (Fig. 5i), validating that miR-654-3p could target SYTL2 in spinal OS. Then favorable knockdown efficiency of SYTL2 was gained in U2OS and Saos-2 cells (Fig. 5j). And by application of CCK-8 and colony formation assays, cell proliferation was depressed by inhibition of SYTL2 (Fig. 4k, l). Cell apoptosis was induced by SYTL2 silence (Fig. 4m, n). Cell migration and invasion were hampered by SYTL2 attenuation (Fig. 4o, p). Furthermore, AKT and ERK pathways were not influenced by SYTL2 knockdown (Additional file 1: Fig. S1E). To sum up, SYTL2 acted as the downstream target gene of miR-654-3p and could facilitate spinal OS development.

**Fig. 6:**
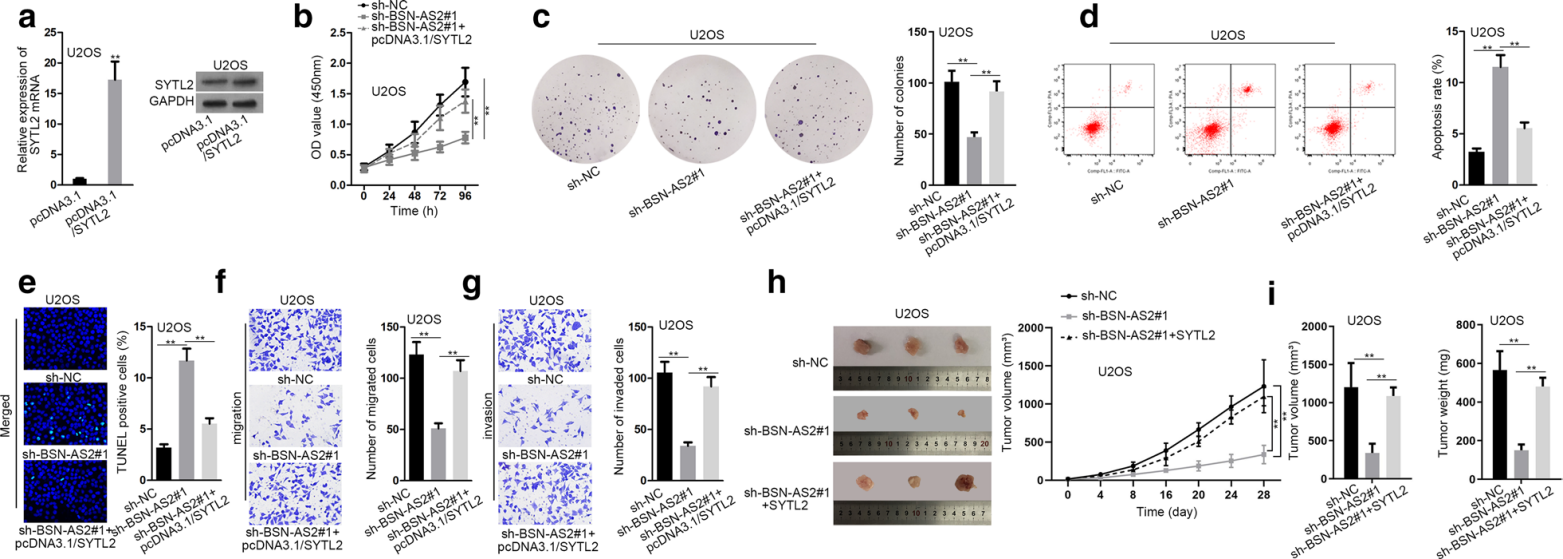
BSN-AS2 accelerated spinal OS progression in a SYTL2-dependent way. **a** qRT-PCR and western blot assays examined SYTL2 mRNA and protein levels. **b**, **c** CCK-8 and colony formation assays illustrated cell proliferation in different groups. **d**, **e** Flow cytometry analysis and TUNEL assay evaluated cell apoptosis in transfected U2OS cells. **f**, **g** Transwell assay observed cell migration and invasion in transfected cells. **h** The photos of tumor obtained from mice in differently transfected groups were taken. Tumor volume curve was depicted. **i** Tumor volume and weight were measured. ^**^P < 0.01

Finally, rescue experiments were performed in U2OS cells and SYTL2 was overexpressed for the experiments using pcDNA3.1/SYTL2 vector. Obviously, SYTL2 expression was strongly increased in U2OS cells (Fig. 6a). SYTL2 upregulation could reverse the inhibitory effect on cell proliferation caused by BSN-AS2 silence (Fig. 6b, c). Elevated cell apoptosis by BSN-AS2 downregulation was obstructed by SYTL2 overexpression (Fig. 6d, e). Oppositely, cell migration and invasion inhibited by BSN-AS2 depression were respectively rescued by SYTL2 upregulation (Fig. 6f, g). Meanwhile, the regulation of BSN-AS2 and YTL2 expressions didn’t affected the AKT and ERK pathways (Additional file 1: Fig. S1F). Additionally, in vivo studies were carried out to further validate above findings. As a result, tumors had a slower growth rate in BSN-AS2-slineced groups, resulting in smaller volume and lighter weight when being excised from mice (Fig. 6h, i). On the whole, SYTL2 was involved in the BSN-AS2-mediated spinal OS progression.

## Discussion

Spinal osteosarcoma (OS) is an aggressive malignancy with a poor outcome. Hence, it is imperative to identify novel therapeutic targets for spinal OS. LncRNAs locating in cytoplasm could function as competing endogenous RNAs (ceRNAs) to sponge microRNAs (miRNAs) and regulate gene expression, thus influencing cancer progression. For example, STAT3-induced lncRNA HOXD-AS1 facilitates liver cancer metastasis by competitively sponging miR-130a-3p and regulating SOX4 [27]. LncRNA NEAT1 sponges miR-129 to regulate the progression of esophageal squamous cell carcinoma via increasing CTBP2 expression [28]. LncRNA SNHG5 serves as a sponge of miR-32 to promote proliferation and migration of gastric cancer cells via targeting KLF4 [29]. Nonetheless, whether BSN-AS2 acted as a ceRNA in spinal OS remained to be explored. In present study, BSN-AS2 expression was confirmed to be elevated in spinal OS tissues and cell lines relative to control groups. Then we found that transcription factor E2F1 could bind with BSN-AS2 promoter to enhance the transcription of it. In addition, BSN-AS2 deficiency obviously suppressed cell proliferation, migration and invasion as well as evidently enhanced cell apoptosis in spinal OS. Moreover, E2F1 was positively correlated with BSN-AS2. Taken together, BSN-AS2 transcriptionally activated by E2F1 facilitated the progression of spinal OS.

To date, mounting miRNAs have been confirmed as key factors in the development of cancer. Abnormally expressed miRNAs have been shown to be implicated in the carcinogenesis of several cancers. For example, miR-92a-3p is sponged by MT1JP to regulate the progression of gastric cancer [30]. MiR-146b-5p aggravates papillary thyroid carcinoma cell migration and invasion and has a positive correlation with the degree of malignancy [31]. MiR-188-5p inhibits cell proliferation and metastasis via targeting FGF5 in hepatocellular carcinoma [32]. In current study, miR-654-3p was potentially sponged by BSN-AS2. It has reported that inhibition of miR-654-3p accelerates the progression of papillary thyroid cancer [33]. However, the interaction between BSN-AS2 and miR-654-3p in spinal OS needed to be further investigated. In our study, BSN-AS2 and E2F1 negatively regulated miR-654-3p in spinal OS cells. As expected, miR-654-3p had negative correlation with BSN-AS2 about the expression in in spinal OS tissues. Meanwhile, miR-654-3p overexpression blocked the development of spinal OS progression. In summary, miR-654-3p was sequestered by BSN-AS2 and could suppress spinal OS progression.

Synaptotagmin-like protein 2 (SYTL2) was previously identified to increase the metastatic potential of ovarian cancer [34]. In this study, bioinformatics analysis indicated SYTL2 served as a downstream target of miR-654-3p. Upregulation of miR-654-3p or shortage of BSN-AS2 could decrease the expression of SYTL2. Of note, E2F1 overexpression could stimulate SYTL2 expression. There existed negative/positive correlation between SYTL2 and miR-654-3p/BSN-AS2. Besides, SYTL2 knock down inhibited the progression of spinal OS. At last, rescue assays illustrated that enforced expression of SYTL2 largely abolished the BSN-AS2 deficiency-induced inhibitory influences on the development of spinal OS.Our work elucidated a ceRNA network constructed by BSN-AS2/miR-654-3p/SYTL2 in spinal OS.

## Conclusion

E2F1-activated BSN-AS2 contributed to spinal OS progression by targeting miR-654-3p/SYTL2 axis, revealing a new perspective into understanding spinal OS.

## Supplementary information

**Additional file 1: Fig. S1.** (A) Western blot assays measured the expression of AKT/ERK pathway-related proteins when silencing BSN-AS2. (B) RNA pull down assays tested the enrichment of the indicated miRNAs in biotin BSN-AS2 probe group. (C–F) Western blot assays measured the expression of AKT/ERK pathway-related proteins under different transfection conditions. ^*^P < 0.05, ^**^P < 0.01. n.s. represented no significance.

## Data Availability

Research data and material are not shared.

## Abbreviations

OS: Osteosarcoma
lncRNAs: Long non-coding RNAs
qRT‐PCR: Quantitative Real-Time PCR
ceRNAs: Competing endogenous RNAs
miRNAs: MicroRNAs
SYTL2: Synaptotagmin-like protein 2
