# Supplementary figures and images for: Long noncoding RNA BSN-AS2 induced by E2F1 promotes spinal osteosarcoma progression by targeting miR-654-3p/SYTL2 axis
Source: Cancer Cell Int. 2020 Apr 25;20:133. doi: 10.1186/s12935-020-01205-y (PMC7183609; doi:10.1186/s12935-020-01205-y)

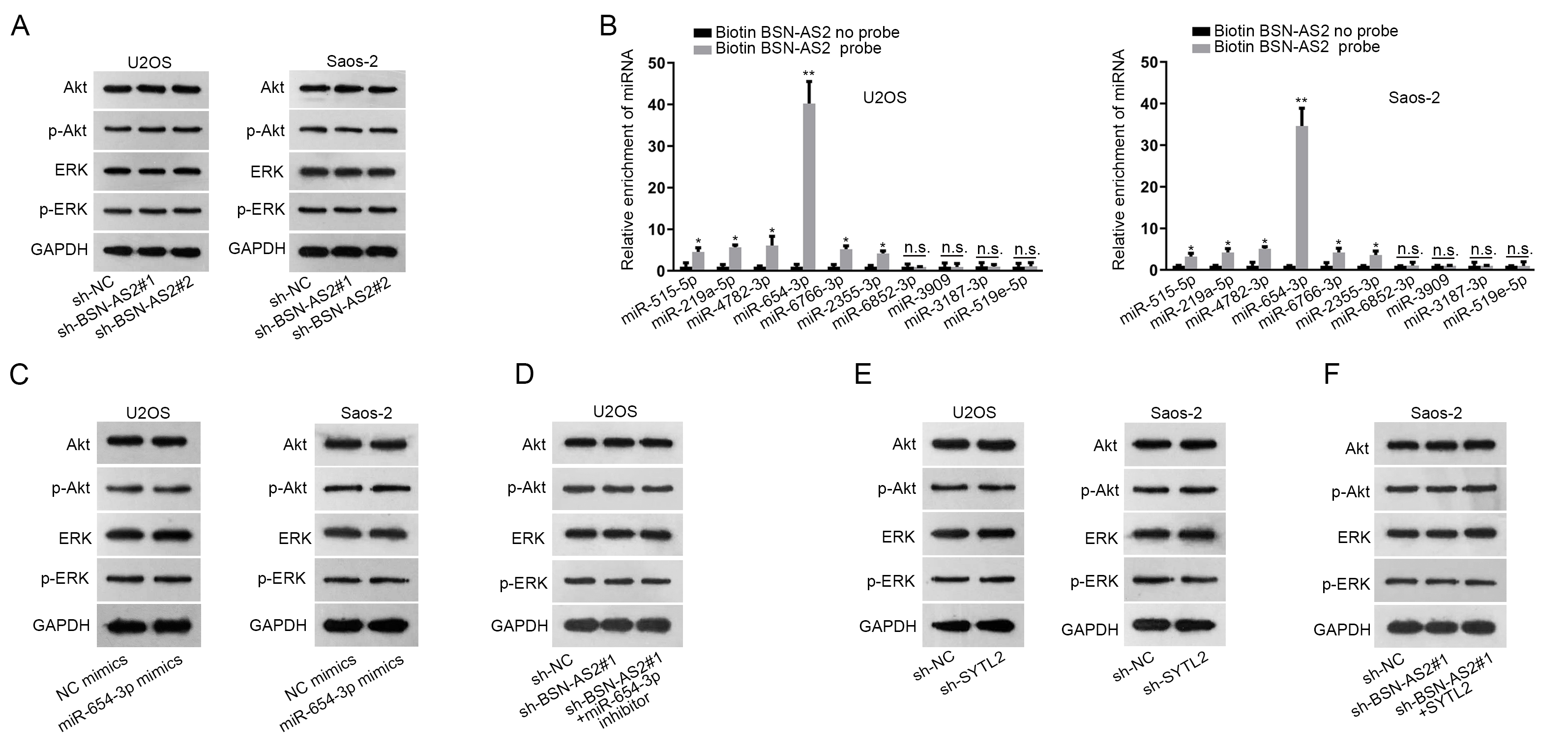

Supplement: Supplementary file 1 — Additional file 1: Fig. S1. (A) Western blot assays measured the expression of AKT/ERK pathway-related proteins when silencing BSN-AS2. (B) RNA pull down assays tested the enrichment of the indicated miRNAs in biotin BSN-AS2 probe group. (C–F) Western blot assays measured the expression of AKT/ERK pathway-related proteins under different transfection conditions. *P < 0.05, **P < 0.01. n.s. represented no significance. [file 12935_2020_1205_MOESM1_ESM.tif]
